# Identification of a protein expression signature distinguishing early from organising diffuse alveolar damage in COVID-19 patients

**DOI:** 10.1136/jcp-2023-208771

**Published:** 2023-03-09

**Authors:** Helen Ashwin, Luke Milross, Julie Wilson, Joaquim Majo, Jimmy Tsz Hang Lee, Grant Calder, Bethany Hunter, Sally James, Dimitris Lagos, Nathalie Signoret, Andrew Filby, Omer Ali Bayraktar, Andrew J Fisher, Paul M Kaye

**Affiliations:** 1 York Biomedical Research Institute, Hull York Medical School, University of York, York, UK; 2 Newcastle University Translational and Clinical Research Institute, Newcastle upon Tyne, UK; 3 Department of Mathematics, University of York, York, UK; 4 Department of Cellular Pathology, Newcastle Upon Tyne Hospitals NHS Foundation Trust, Newcastle Upon Tyne, UK; 5 Wellcome Sanger Institute, Wellcome Genome Campus, Hinxton, UK; 6 Biosciences Technology Facility, University of York, York, UK; 7 Biosciences Institute, Newcastle University, Newcastle upon Tyne, UK; 8 Institute of Transplantation, Newcastle Upon Tyne Hospitals NHS Foundation Trust, Newcastle Upon Tyne, UK

**Keywords:** COVID-19, LUNG, INFLAMMATION, Immune System Diseases

## Abstract

Diffuse alveolar damage (DAD) is the histological expression of acute respiratory distress syndrome and characterises lung pathology due to infection with SARS-CoV-2, and other respiratory pathogens of clinical significance. DAD reflects a time-dependent immunopathological process, progressing from an early/exudative stage through to an organising/fibrotic stage, yet within an individual these different stages of DAD may coexist. Understanding the progression of DAD is central to the development of new therapeutics to limit progressive lung damage. Here, we applied highly multiplexed spatial protein profiling to autopsy lung tissues derived from 27 patients who died from COVID-19 and identified a protein signature (ARG1, CD127, GZMB, IDO1, Ki67, phospho-PRAS40 (T246) and VISTA) that distinguishes early DAD from late DAD with good predictive accuracy. These proteins warrant further investigation as potential regulators of DAD progression.

## Introduction

The COVID-19 pandemic has claimed over 6.6 million lives and despite vaccines that prevent serious illness and use of dexamethasone in severely ill patients, worldwide deaths continue to accrue.^1^ There is, therefore, a continued need to identify new treatment options to minimise disease severity. Previous analyses of autopsy cases have identified diffuse alveolar damage (DAD) as a primary histological feature associated with fatal COVID-19.^2 3^

Based on the analysis of autopsy cases that have succumbed to infection over different time periods, DAD is often represented as a continuum of immunopathology. Soon after tissue insult, early or exudative DAD (EDAD) is characterised by hyaline membrane, fibrin extravascation into the alveoli, pulmonary oedema and the presence of focal interstitial infiltration. Progression to organising DAD (ODAD) is associated with loose areas of fibrosis and commonly chronic interstitial inflammation, whereas in the final stage, fibrotic DAD, dense collagen and thickening of alveolar walls is observed. To date, however, formal evaluation of protein markers associated with inflammation during progression through these stages of DAD have been limited to low-plex immunohistochemistry studies of specific immune cells^4^ or have focused on the alveolar epithelium.^5^ Broader transcriptomic analyses including spatial transcriptomics have indicated the heterogeneity in the lung response in COVID-19 patients^6 7^ but have not specifically compared DAD at different stages of progression. Thus, proteins associated with progression from EDAD to ODAD remain unclear, a knowledge gap that represents a roadblock to the identification of new therapeutic agents able to prevent progression to pulmonary fibrosis.

To address this question, we examined lung tissue from a cohort of COVID-19 autopsy cases in the UK. We used digital spatial profiling (DSP) to determine differences in protein expression between regions of interest (ROI) identified histologically as EDAD or ODAD. We focused on protein targets with therapeutic potential demonstrated in other diseases and/or preclinical models to identify potential regulators of DAD progression with potential to be rapidly translated in the clinic through drug repurposing.

## Materials and methods

### Patient samples

Lung tissue from 27 patients (5 female, 22 male; 7 black/Asian/minority ethnic, 20 caucasian) who had died with SARS-CoV-2 during the first and second wave of the pandemic were selected from a larger cohort assembled by the UK Coronavirus Immunology Consortium (UK-CIC). Prior to death, 7/27 were known to have received steroids, 19/27 antibiotics and 13/27 anticoagulants. None to our knowledge had received treatment with antivirals. Median time from death to post mortem was 3 days (range 1–9 days) (online supplemental table S1). A full description of the UK-CIC cohorts will be provided elsewhere (Milross *et al*, ms in preparation). Patients were selected for the current study based on histological evidence of DAD without concurrent bronchopneumonia or histology attributable to acute cardiac failure. ROIs (approx. 600 µm^2^) reflecting EDAD, ODAD or a mixed phenotype (MDAD) were identified by a pathologist with cardiothoracic expertise on H&E-stained formalin fixed paraffin embedded (FFPE) sections and used to guide subsequent ROI selection for protein spatial profiling. Patient data relating to pandemic wave, ethnicity, age, sex, illness duration and place of death are provided in online supplemental table S1.

10.1136/jcp-2023-208771.supp1 Supplementary data

### Nanostring GeoMx protein spatial profiling

The 4 µm thick FFPE lung sections were used for protein spatial profiling using the Nanostring GeoMx platform. Slides were stained with CD3 and CD68 as morphological markers and with a panel of 68 oligo-nucleotide conjugated antibodies comprising the Immune Cell Profiling Core (24 Abs), IO Drug Target Panel (10 Abs), Immune Activation Status Panel (8 Abs), Immune cell Typing Panel (7 Abs), PI3K/AKT Signalling Panel (9 Abs) and the MAPK Signalling Panel (10 Abs). Regions conforming the histological description of EDAD and ODAD were identified in each patient’s lung tissue. ROI capture was performed using a GeoMx Spatial profiler instrument (Nanostring, Seattle, Washington, USA).

Digital count data were normalised to positive ERCC controls and to housekeeping controls (GAPDH and Histone H3). Housekeeping targets were selected based on high correlation with isotype controls. ROIs with abnormal levels of hybridisation, HK expression or low isotype control background were removed from the analysis. Proteins were thresholded from analysis if signals were below the geometric mean of the isotype controls in >90 of ROIs. Data for the 40 proteins passing QC and thresholding (online supplemental table S1) were exported for further analysis in R (see Statistical Analysis) and analysed using linear mixed modelling using GeoMx software (V.2.0) with patient ID and cohort selected as random variables. Data were analysed using GeoMx software to generate significance scores with false discovery rate (FDR) correction (5%) based on Benjamini, Krieger and Yekutieli two stage setup method and Log2 fold change cut-off of 0.589 (1.5-fold change) between pathology classes. Volcano plots were generated in GraphPad Prism (V.9.1).

### Statistical analysis

Statistical analyses were carried out in R V.4.1.1.^8^ The base R function prcomp was used for principal components analysis (PCA), while the pls package^9^ was used for partial least squares regression (PLS-R). Classification was performed using the plsgenomics R package^10^ with leave-one-patient-out (LOPO) cross-validation to avoid overfitting in this supervised approach. Here, all ROIs for each patient in turn were left out and the remaining data used to build the model which was then used to predict the class of the left-out ROIs. Results are shown for the ROIs that were not used in model training. In order to show the predictive accuracy as the discriminatory threshold was varied, a receiver operating characteristic (ROC) was generated using the R package ROCR.^11^

## Results

**Figure 1:**
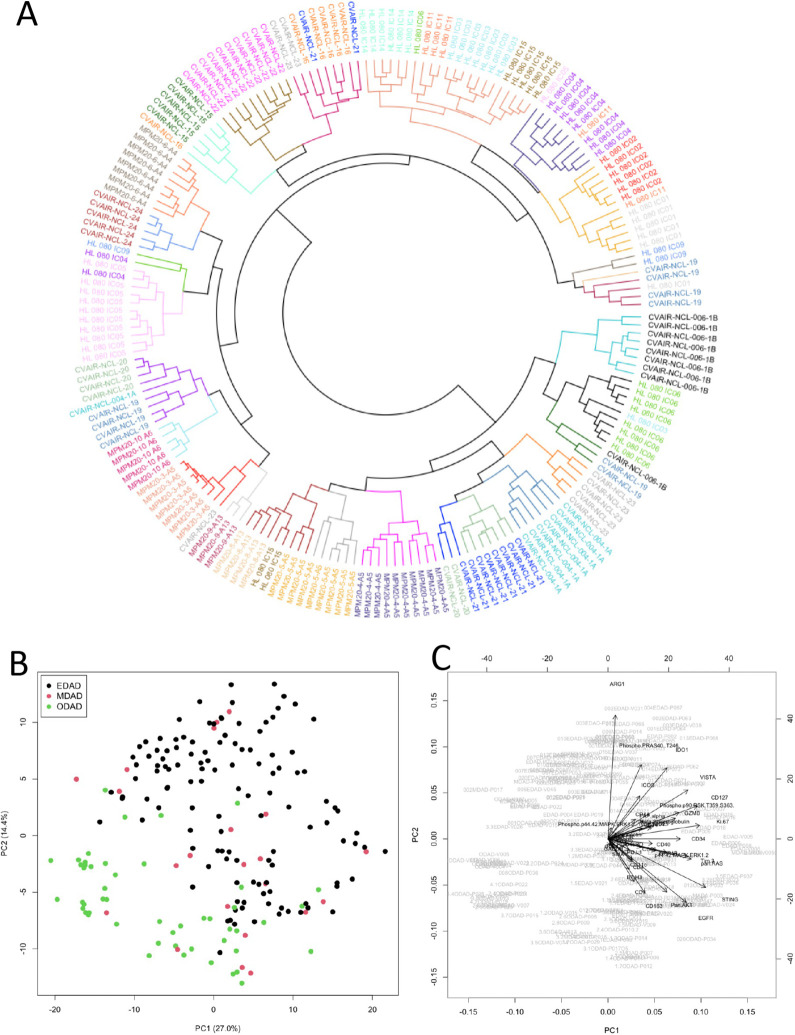
Analysis of protein DSP data. (A) Circular dendrogram from hierarchical clustering of protein DSP ROIs (with patient identifiers colour-coded and clusters coloured separately; see online supplemental table S1. (B, C) Principal component analysis (PCA) scores plot for the first two principal components coloured by EDAD, MDAD and ODAD (B) and with loadings shown as vectors (C). DSP, digital spatial profiling; EDAD, exudative DAD; MDAD, mixed DAD; ODAD, organising DAD; ROI, regions of interest.

**Figure 2:**
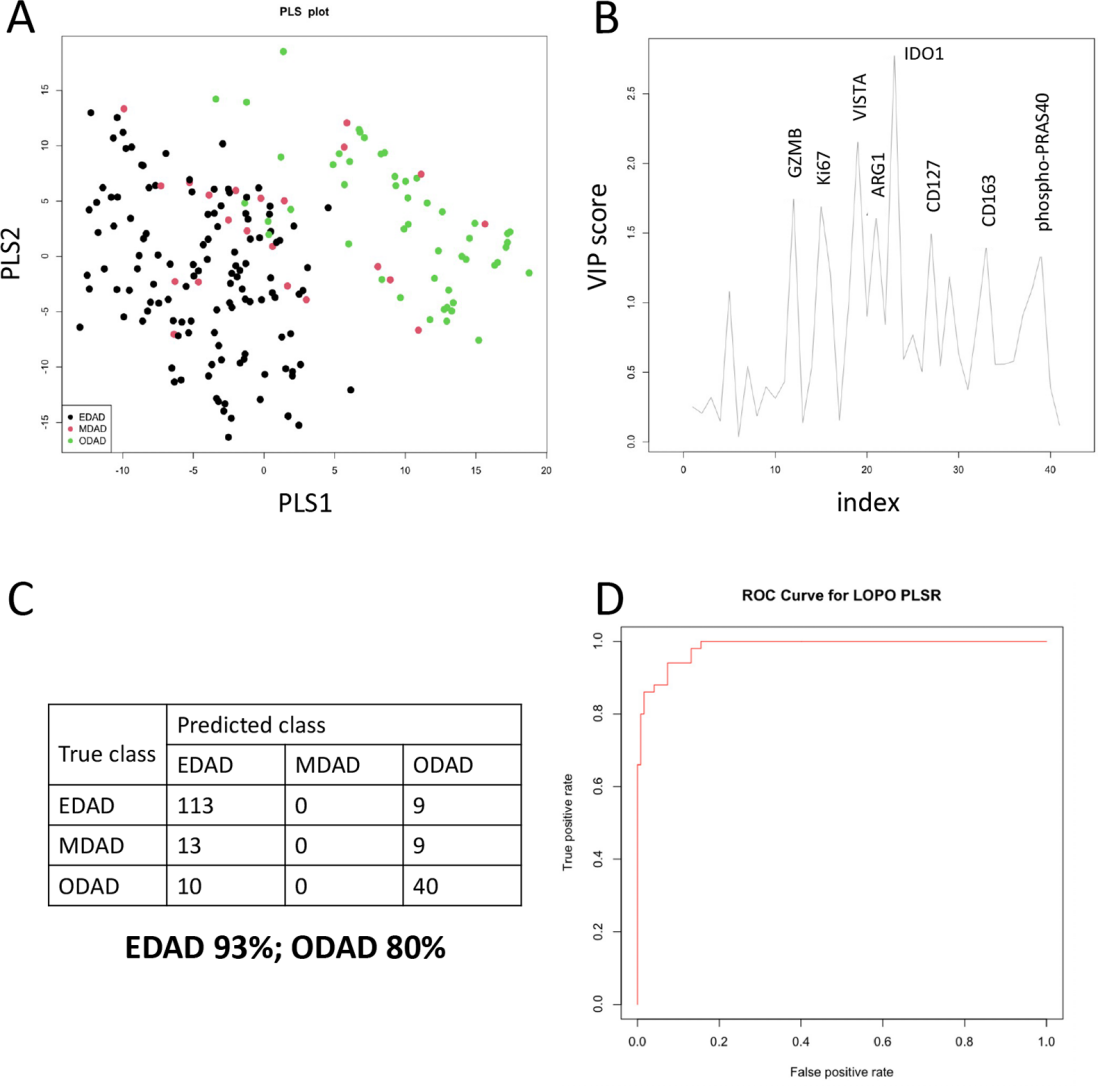
Discrimination of DAD classes based on protein signature. (A, B) Partial least squares analysis of EDAD, MDAD and ODAD samples shown as PLS plot (A) and by variable importance in projection (VIP) score (B). (C) Confusion matrix for results of PLS-LDA leave one patient out prediction using eight variables with VIP scores >1.3 (GZMB, Ki.67, VISTA, ARG1, IDO1, CD127, CD163, Phospho.PRAS40). (D) Receiver operating characteristic (ROC) curve generated for EDAD versus ODAD ROIs. DAD, diffuse alveolar damage; EDAD, exudative DAD; LDA, linear discriminate analysis; MDAD, mixed DAD; ODAD, organising DAD; PLS, partial least square; ROI, regions of interest.

We examined 194 ROIs (7±2 ROIs per patient; 122 EDAD, 50 ODAD, 22 MDAD; online supplemental figure S1) for the expression of 40 proteins using GeoMx DSP. As anticipated, we generally observed clustering of ROIs by patient, reflecting repeat sampling (figure 1A and online supplemental table S1). PCA showed separation of each form of DAD with 41.4% of variance accounted for by PC1 and PC2 (figure 1B,C). We next applied PLS-R (figure 2A) and identified variables responsible for group separation using variable importance in projection (VIP) scores. Proteins with VIP scores >1.3 (ARG1, CD127, CD163, GZMB, IDO1, Ki67, phopsho-PRAS40 (T246) and VISTA; figure 2B) largely mirrored what was observed with PCA (figure 1C). These eight variables were used to classify ROIs in PLS linear discriminate analysis with LOPO cross-validation to prevent overfitting. This achieved a predictive accuracy of 93% and 80% for EDAD and ODAD, respectively (figure 2C). MDAD ROIs were consistently misclassified, likely a reflection of heterogeneity and the transitional nature of the pathology within this group. Finally, we generated an ROC curve for EDAD and ODAD samples using LOPO cross-validation in PLS-R, showing the predictive accuracy as the discriminatory threshold is varied (figure 2D).

10.1136/jcp-2023-208771.supp2 Supplementary data

**Figure 3:**
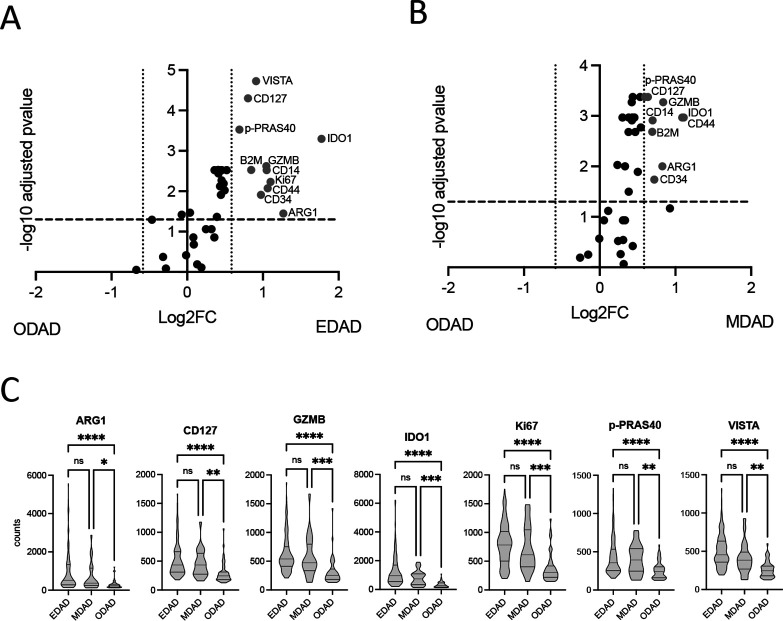
Differential target expression between EDAD and ODAD using linear mixed modelling. (A, B) Differentially expressed (FDR 5%; FC=1.5) protein targets between EDAD and ODAD (A) and MDAD and ODAD (B). Data derives from a linear mixed modelling with patient repeat measures and cohort as a random effect. (C) Individual ROI counts for EDAD, MDAD and ODAD ROIs for identified target proteins. *p<0.05, **p<0.01, ***p<0.001, ****p<0.0001 between indicated groups. ns, non-significant; EDAD, exudative DAD; MDAD, mixed DAD; ODAD, organising DAD; ROIs, regions of interest. FDR, false discovery rate.

We independently analysed these data using linear mixed modelling to account for potentially confounding factors (including repeat measures and cohort effects) and identified eleven targets (ARG1, B2M, CD14, CD34, CD44, CD127, GZMB, IDO1, Ki67, phospho-PRAS40 (T246) and VISTA) distinguishing EDAD and ODAD (>1.5 fold change cut-off, FDR=5%; figure 3A,C). MDAD was similarly distinguished from ODAD (figure 3B,C), but no proteins were significantly different between EDAD and MDAD.

Collectively, our data suggest a core protein signature comprising ARG1, CD127, GZMB, IDO1, Ki67, phospho-PRAS40 (T246) and VISTA distinguishes EDAD from ODAD ROIs in this patient group. Nevertheless, our data also suggest further patient heterogeneity within EDAD ROIs. This was most marked for ARG1, which was absent from all EDAD ROIs in 8/20 patients. Although sample size precluded a formal analysis, this appeared unrelated to sex, place of death, duration of disease, cohort or prior treatment (online supplemental table S1).

## Discussion

Using DSP to interrogate well-annotated lung tissue, we identified a core protein signature discriminating early from late phases of DAD. Not surprisingly given the targeted nature of our panel, the proteins we identified have well-known functions in inflammation and immunity, but they have not previously been evaluated in relation to DAD progression. ARG1 is elevated in the lungs of severe COVID-19 patients, being expressed by CD11b^+^CD66b^+^ granulocytic myeloid-derived suppressor cells.^12^ IDO1 has been detected in lung tissue in other autopsy series,^13 14^ and due to its broad expression on endothelial cells, has been implicated in the vasodilation/vasoplegia associated with initial stages of COVID-19 pneumonia.^14^ CD127 expression on monocytes has been noted at sites of hyperinflammation,^15^ whereas VISTA has been proposed as a therapeutic target to minimise inflammation.^16^ A single study found there was a trend for higher GZMB expression to be associated with DAD in patients receiving allogeneic lung transplants.^17^

Finally, phosphorylation of PRAS40 at T246 releases mTORC1 to perform its many downstream functions and elevated phopsho-PRAS40 (T246) has been used as a biomarker of PI3K/Akt/mTORC1 activation,^18^ a pathway implicated in idiopathic pulmonary fibrosis and DAD.^19^ While mechanistic studies are required in preclinical models to confirm causality, and possible changes in cellularity need to be considered, the heightened expression in EDAD of proteins associated with mononuclear phagocyte activation suggests that the EDAD-ODAD transition may be associated with dampening of a hyperinflammatory state.

This study has limitations: (1) DSP quantifies protein expression across the entire ROI and cannot distinguish multiple cells with low target expression vs few cells with high target expression; (2) Our patient cohort was too small to perform sub-group analysis based on age, gender, disease duration, place of death or prior treatment; (3) We cannot rule out that patients had other forms of concurrent disease or different forms of DAD in other areas of lung not sampled here and this may account for some of the inter-patient heterogeneity observed; (4) Further validation is required in an independent patient cohort, preferably incorporating single cell technologies.

Notwithstanding these limitations, to our knowledge, this is the first study to apply highly multiplexed DSP to discriminate between EDAD and ODAD. The extent to which the many millions of COVID-19 survivors are at risk of developing pulmonary fibrosis is only beginning to be understood.^20^ Importantly, many of the protein targets we have identified as being highly expressed at the early stages of DAD are amenable to therapeutic intervention with existing drugs or drugs in development. Hence, further exploration of these targets as potential regulators of DAD progression using preclinical models of SARS-CoV-2, as well as in other diseases associated with DAD, could provide an evidence base on which to conduct future intervention trials.
