# Supplementary material for: Identification of a protein expression signature distinguishing early from organising diffuse alveolar damage in COVID-19 patients
Source: J Clin Pathol. 2023 Mar 9;76(8):561–5. doi: 10.1136/jcp-2023-208771 (PMC10423525; doi:10.1136/jcp-2023-208771)
Supplement: Supplementary data [file jcp-2023-208771supp002.pdf]

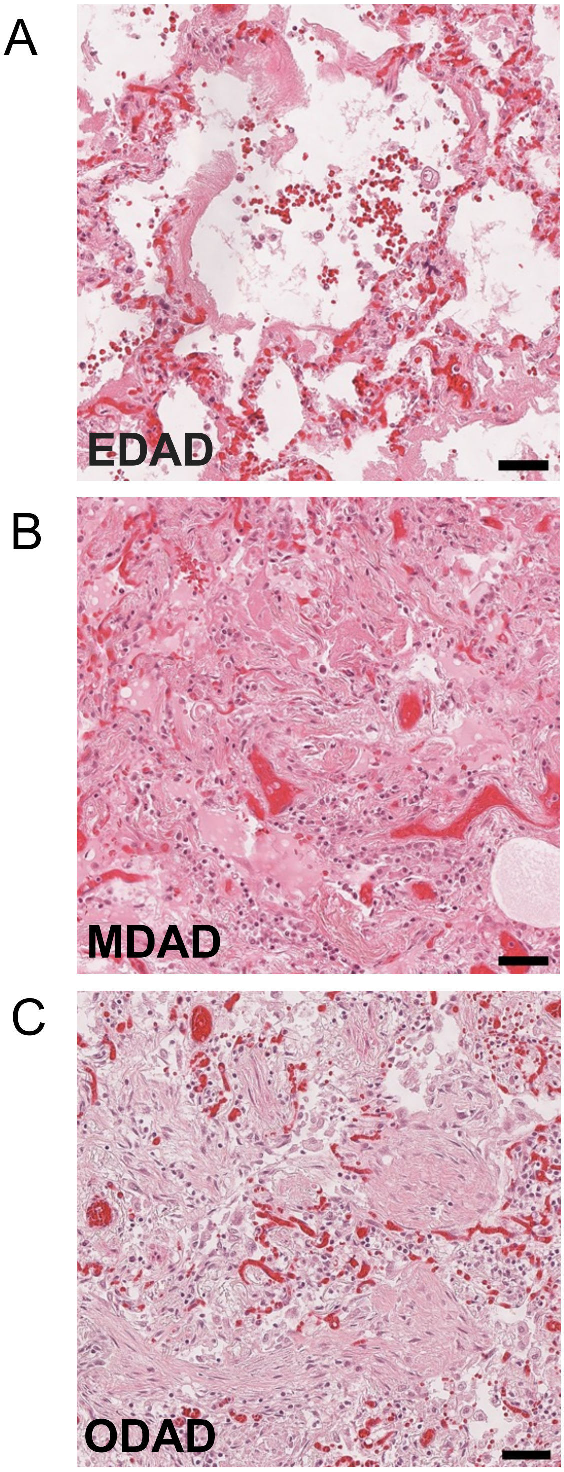

Figure S1. Representative images showing DAD progression
A) Exudative DAD (EDAD). **B**) mixed DAD (MDAD). **C**) organising DAD (ODAD).
Scale bar = 50µm
